# Arduino-Based Low-Cost Device for the Measurement of Detonation Times in Blasting Caps

**DOI:** 10.3390/s23146534

**Published:** 2023-07-19

**Authors:** Eduardo Cámara-Zapata, Arnau Arumi-Casanovas, Jordi Bonet-Dalmau, Marc Bascompta, Lluís Sanmiquel

**Affiliations:** Department of Mining, Industrial and ICT Engineering, Polytechnic University of Catalonia (UPC), Manresa, Av. Bases de Manresa, 61-73, 08242 Barcelona, Spain; eduardo.camara@upc.edu (E.C.-Z.); arnau.arumi@upc.edu (A.A.-C.); jordi.bonet@upc.edu (J.B.-D.); lluis.sanmiquel@upc.edu (L.S.)

**Keywords:** blasting caps, scattering, Arduino-based, high-precision device, low-cost system, drift correction

## Abstract

The use of equipment such as oscilloscopes, high-speed cameras or acoustic sensors is quite common to measure detonation times from surface connectors and detonators. However, these solutions are expensive and, sometimes, not adequate to use in field conditions, such as mining or civil works. In this regard, a low-cost portable device is designed and tested using the Arduino platform, achieving a simple, robust and precise system to carry out field measurements. This study describes the characteristics and working principles of the designed device, as well as the verifications carried out to check the accuracy of the Arduino ceramic oscillator. Additionally, a field test was carried out using 100 actual detonators and surface connectors to verify the correct operation of the designed equipment. We have designed a device, and a methodology, to measure detonation instants with a minimum accuracy of 0.1 ms, being sufficient to carry out subsequent studies of detonation time dispersion for non-electric detonators.

## 1. Introduction

The use of explosives for excavations is a widely used technique in mining and civil works, obtaining the effective and economical removal of rocks [1]. Blasting is based on the drilling of a mesh of holes in which the explosive is introduced, achieving an appropriate distribution of the energy contained in the explosive within the volume of rock to be blasted [2,3].

Blasting is initiated using detonators, providing the required activation energy to the explosive in the blast hole [4]. These detonators can be electric, non-electric or electronic. The detonation of each blast hole must occur in an established order and at certain time intervals, achieving it using micro-delay detonators [1,3,5,6]. Electric and non-electric blasting caps contain a pyrotechnic delay element that will give the time delay according to its length and reaction speed, acting similarly to a fuse, while electronic detonators contain a microchip that allows programing the detonation time with high accuracy [7].

Blasting caps with pyrotechnic delay have a dispersion in the detonation times with respect to their nominal values due to the chemical nature of the delay element itself, whose reaction speed can suffer slight variations due to aging of the delay pyrotechnic element, density/porosity variation due to production, small changes in its composition, temperature changes, different manufacturing batches, etc. [8]. Dispersion values of pyrotechnic delay detonators are given in several publications [7,9,10,11], usually being a dispersion between 4 to 8% of their nominal value.

If the detonation of a blast hole occurs at an instant far from its nominal value, overlapping or detonations outside the expected time can occur, with the appearance of adverse effects such as projections, vibrations, airwaves, poor fragmentation, poor blast performance, etc. [12,13,14,15]. Hence, it is crucial to know the actual instant of detonation of the detonators in order to study the accuracy of the initiation system and its influence on the blasting results. For this purpose, commercial equipment can be used to measure the blast holes’ moment of detonation, for instance, the equipment that allows monitoring blast holes to measure the detonation velocity (VoD) of the explosive [16,17] and obtain, as additional information, the detonation time delay between each blast hole. Another alternative is the use of high-speed cameras for ultra-slow motion image analysis. However, this last option is quite expensive, often out of reach for small operations and/or extracting materials of low economic value, and the potential issues previously mentioned remain present.

The main goal of this study is to design a low-cost, portable and easy-to-use device, based on the Arduino platform, to measure the detonation instants of detonators with an accuracy of at least 0.1 ms. We describe the equipment proposed, its operating principle and the correction of the drift in the microcontroller clock. Finally, field measurements were recorded to verify the functioning and accuracy of the new device.

## 2. Detonation Time Measurement from Detonators

There are several alternatives to measure the detonation times from detonators and surface connectors, each one with advantages and disadvantages, gathered in the following subsections.

### 2.1. Acoustic Sensors

Verna et al. [10] measured the time delay of electric detonators, recording the instant of the pulse of the ignition current applied to the detonator and the detonation instant using a microphone. The system is simple, but it has the disadvantages that each detonator must be tested individually in a laboratory and it is not suitable for non-electric detonators and surface connectors. In addition, the acoustic sensor is placed very close to the detonators, only 0.8 m away, so its use in real blasting is ruled out due to the high risk of equipment breakage.

An electro-acoustic sensor was also used by Pytlik et al. [18] to measure detonation times between two connectors or detonators, which may be non-electric. The detonators have to be placed at the same distance from the acoustic sensor to avoid systematic errors in the time taken for the sound wave to reach the sensor. As in the previous case, this system is not applicable to real production blasting, since it requires the same distance between detonators and the sensor. Besides, the acoustic signal may be masked by wind or external noises, among other factors, together with the risk of equipment breakage due to its proximity to the blast.

### 2.2. VoD Measurement Equipment

Commercial equipment is available to measure the velocity of detonation (VoD) of an explosive, based on the variation of the electrical resistance of a wire probe inserted into a blast hole as it is destroyed by the blast [16]. The detonation time delay from bore to bore can be obtained as a by-product of the VoD recordings.

Commercial VoD equipment has been used to measure the detonation times of electronic detonators [18]. However, the measurements fail in 70% of the cases, because the detonators are not powerful enough to break the special probe wire designed to be consumed by the explosion inside a blast hole. This fact makes the system not suitable to measure the detonation moments of surface connectors, whose power and explosive charge are much lower than that of bottom-of-hole detonators.

### 2.3. High-Speed Camera

Another alternative to measuring the detonation times of surface connectors and detonators is the use of high-speed cameras, analysing images in slow motion and visually determining the frames in which detonation occurs. This system is used to simultaneously measure dozens of detonators of any type [18], as long as it is in the camera’s field of view and the camera has enough internal memory to record the entire sequence. Additionally, the equipment is expensive and it must be located in a safe place to avoid damage from detonator shrapnel.

While this system may be valid to determine the detonation time of surface connectors in a real blast, it is not suitable to measure the detonation instant of bottom-of-hole detonators, as the event occurs inside the blast hole and is not visible.

### 2.4. Oscilloscope: Open-Circuit Probe

The usage principle is the same as the Arduino device, measuring the time intervals between breaks in a signal cable that cause a step change in voltage. An oscilloscope is a device with a high sampling rate, achieving highly accurate measurements. However, they have the disadvantage that as the number of available channels increases, they become more expensive and, sometimes, the sampling rate decreases. It should also be borne in mind that this equipment is delicate and impractical for use in field tests, where there are environmental hash conditions (rain, dust, must, etc.), no power sockets and a certain risk of breakage due to projections of rock fragments from the blast.

Oscilloscopes were used in a laboratory environment to measure the detonation times of detonators in other studies [18,19]. The measurement of the detonation time intervals is performed manually, using the cursor to select the instant of the analog signal at which the voltage drop occurs. Additionally, the measurement of several circuits is a somewhat laborious process.

Table 1 exposes the different characteristics and potential usages of existing measuring techniques, considering the main advantages and disadvantages of each option.

## 3. Device Description

Unlike high-cost commercial devices, such as high-speed cameras, VoD meters or oscilloscopes, the availability of free hardware and software platforms offers a wide range of possibilities for developers to create open-source electronic projects at an affordable cost.

Among these open-source platforms, the Arduino stands out for its popularity [20,21], chosen to build the low-cost device presented in this study. The Arduino platform allows the design of electronic circuits that incorporate a microcontroller, with various digital and analog inputs/outputs, that can interface with various sensors. The fact that both the software and hardware are open-source offers developers the possibility to create projects completely tailored to their needs in an unlimited number of applications [22]. For example, some fields in which Arduino-based applications have been developed are home automation [23], agriculture [24] or energy [25], among many other usages. In the mining sector, there is also a collection of several applications focused on wearable systems, field monitoring systems and autonomous systems [26]. Although there is no previous evidence of the use of Arduino in the field of explosives and blasting, this study confirms the great versatility offered by the platform and, thus, broadens its wide range of applications.

Apart from its cost, another advantage of the equipment is its portability and simplicity, which allows it to easily measure in a production blasting environment, either in surface connectors or in-hole detonators. Moreover, it does not interfere with blast operations and does not require the transfer of explosive material to a laboratory.

### 3.1. Operating Principles

The principle for recording the detonation instant of each detonator is based on the interruption of the conductivity of a circuit when it is broken by the effects of an explosion. The cable breakage causes the digital input, going from a voltage of 5 V (high) to 0 V (low). The instant at which this event occurs is recorded with the microcontroller using its clock.

### 3.2. Hardware

The detonation time measurement equipment consists of an Arduino mega board with an ATmega 2560 microcontroller and a 16 MHz quartz oscillator. The microcontroller has a 256 kB flash memory where the program containing the operating instructions is stored. The board has 54 digital input/output (I/O) terminals, of which a total of six terminals are used for the connection of the measurement circuits. Another six I/O terminals are used to control the liquid crystal display (LCD) that shows system information. Communication with the microSD card for data recording occupies another four I/O terminals. The rest of the digital pins are left free, so the unit could easily be expanded with a larger number of circuits to measure detonation times.

**Figure 1:**
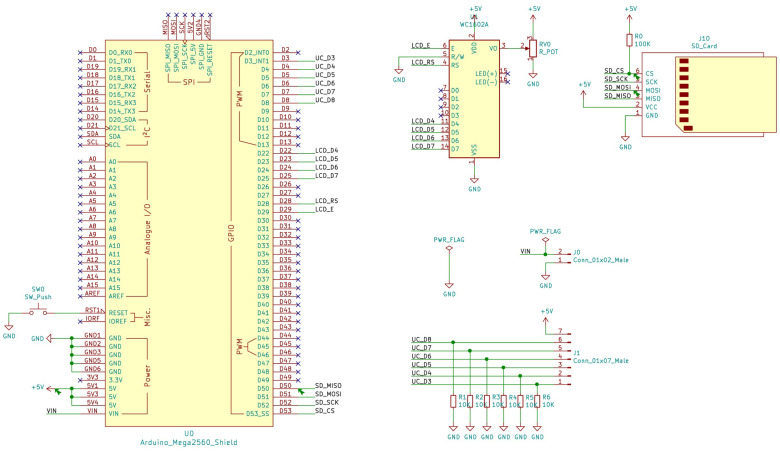
Scheme of the PCB device for the measurement of the detonation time.

To select the unit peripherals and check their operation, an initial prototype has been built through connecting the microcontroller to the rest of the components using a breadboard. This breadboard allows to easily change the wiring and connections, interchange components and test the software until the desired functionality is achieved. Once the peripherals, their connections and the programming of the microprocessor had been validated, a PCB board was designed and manufactured so that all the components could be soldered on it in a solid and resistant way, obtaining a suitable unit to use in field conditions. A scheme of the elements used is shown in Figure 1.

**Figure 2:**
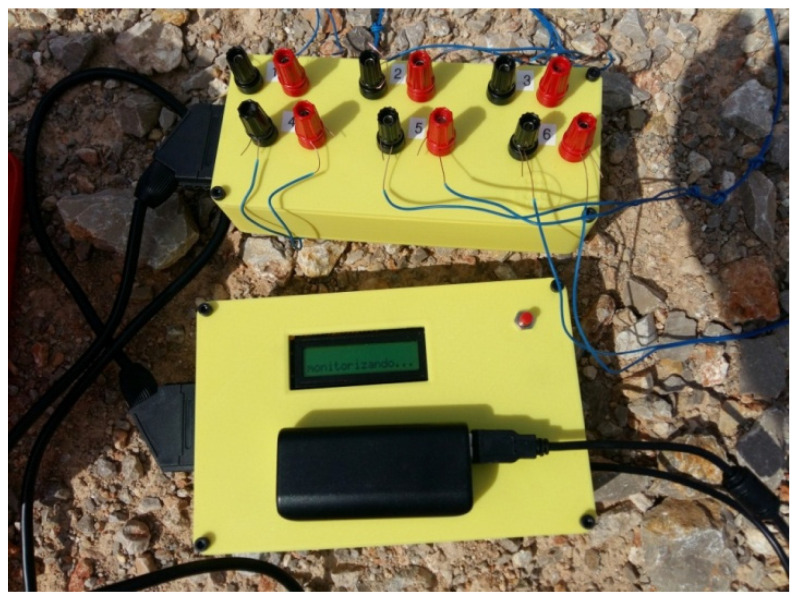
Equipment built. Quick connection box (**upper part**) and microcontroller casing (**lower part**).

The PCB board, with all its components, has been housed inside a custom-made PLA (polylactic acid) thermoplastic casing. Power is supplied by an external rechargeable 5V lithium-ion battery connected to the USB port of the Arduino board. A quick screw connector holder box has been included to facilitate the connection of the measurement circuits to the device. Figure 2 shows the measurement equipment built in one of the tests carried out in the field.

### 3.3. Software

**Figure 3:**
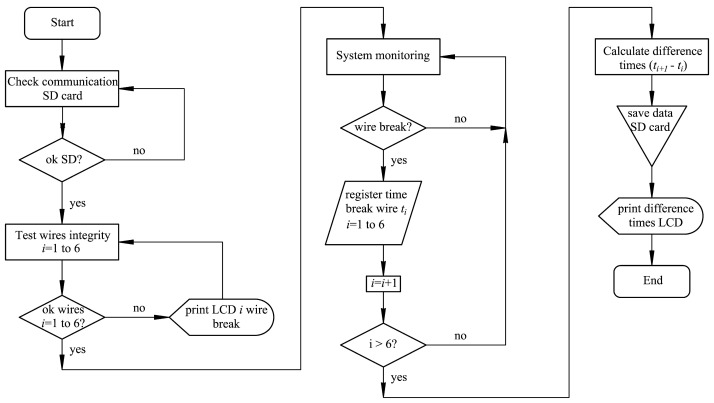
Device driver software flowchart.

The integrated development environment (IDE) was used, being compatible with different platforms (Linux, Windows, Mac) and free to use under the GNU Lesser General Public License. The flowchart of the program that controls the device is shown in Figure 3. The programmed code is attached in Annex 1.

### 3.4. Minimum Interval Measurement Time

The minimum time that must elapse between two events, cable breaks, to be measured is given by the time it takes the microcontroller to execute the code necessary to record the values. To determine this value, the sketch containing the operating instructions has been started with all the measurement circuits in a low state. Under these conditions, the values recorded using the system are 8 or 12 microseconds, i.e., between two and three times the resolution of the function *micros()*, which is 4 microseconds. Therefore, events occurring in a time interval of three times the resolution (0.012 ms) or higher will be recorded without problems, which are the potential blasting conditions.

### 3.5. Minimum Accuracy Required

The minimum accuracy required for statistical studies of detonation time dispersion in non-electric detonators (NONELs) is, at least, 0.1 ms. This accuracy is set in the European standard (EN 13763-16) for the determination of the delay accuracy of detonators and relays [27].

## 4. Functional Validation

The validation of the operation and accuracy of the equipment was performed through preliminary checks, determining the drift of the microcontroller clock and the correction needed to apply to the readings obtained using the device.

The preliminary test consisted of cutting the signal cables with scissors and recording the event using an oscilloscope. This test was carried out to verify the correct functioning of the hardware, the programmed code and the connection protocol between the oscilloscope and the Arduino for the simultaneous recording of the signal. Subsequently, a field test was carried out through firing one hundred detonators in groups of four, recording the analog signal of the cutting of wires, due to the effects of an explosion, using an oscilloscope and the time provided by the microcontroller’s clock, which made it possible to compare both results and validate the correct functioning and accuracy of the equipment.

### 4.1. Clock Drift

**Figure 4:**
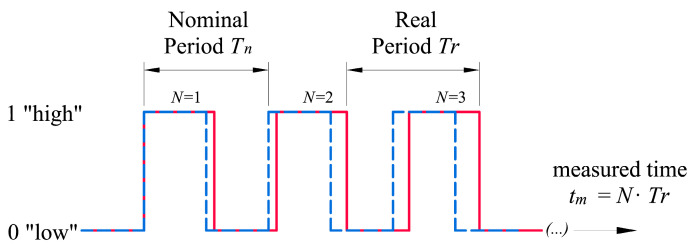
Ideal clock signalas a dashed blue line and real signal as a red line. The real period, T_r_, can be greater or smaller than the nominal period, T_n_, causing a drift, delay or advance of the clock.

Microcontrollers measure time through counting the number of pulses of a periodic signal generated by an oscillator [28], called a clock. Since perfect oscillators do not exist, the signal is affected by an error in the period between pulses, with the actual vibration frequency being different from the nominal one. Apart from this tolerance error, inherent in any manufactured component, the oscillator frequency can also be affected by environmental factors, such as temperature or aging [29]. The difference between actual oscillation frequency and nominal frequency, Figure 4, means that the time measured by the clock has a drift that must be determined and, subsequently, corrected.

The error of a clock controlled by an oscillator of nominal frequency, *f_n_*, relative to an ideal reference time, *t*, is shown in Equation (1) [30].

(1) $$\varepsilon_{t}(t)=\tau_{0}+\left(\phi +M\cdot \left(T^{{}^{\circ}}-T_{ref}^{{}^{\circ}}\right)-1\right)\cdot t+D\cdot t^{2}+\sigma_{x}(t)$$

*e_t_* is the total time error.
*t*_0_ is the initial synchronisation error, i.e., the difference between the time measured by the clock and the reference time.
$\phi =\frac{f_{r}}{f_{n}}$ is the ratio of the actual oscillator frequency, *f_r_*, to the nominal frequency, *f_n_*, at 25 °C.
$\phi =\frac{T_{n}}{T_{r}}$ is the tolerance term. It can also be expressed as a ratio of the nominal oscillator period, *T_n_*, to the actual period, *T_r_*.
*M* is the environmental factor, a frequency-relative variation with respect to temperature.
$T_{ref}^{{}^{\circ}}$ is the reference temperature, usually 25 °C.
*T^°^* is the oscillator temperature.
*D* is the first derivative of the relative variation of the frequency with respect to time, or the ageing term of the oscillator.
*s_x_*(*t*) is the stochastic error term due to signal noise.

**Figure 5:**
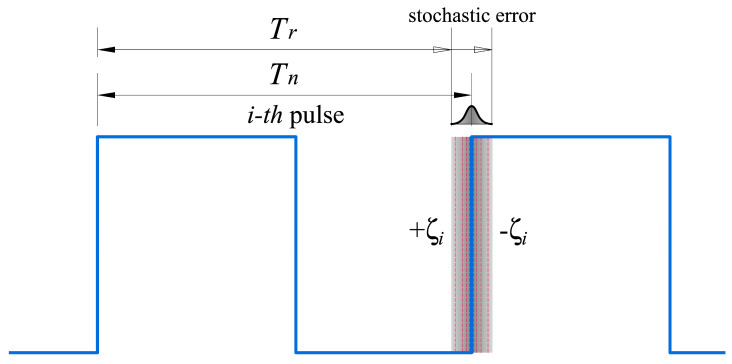
Stochastic error of the *i*-th pulse of a clock due to noise in the signal. *T_n_*—nominal period of the oscillator; *T_r_*—real period of a pulse.

$\sigma_{x}(t)\;=\;\sum_{i=1}^{N}\xi_{i}$, where $\xi_{i}$ is the stochastic error of the i-th pulse of the signal, resulting from the difference between the nominal period, *T_n_*, and the actual period, *T_r_* (Figure 5).

The first three terms from Equation (1) correspond to systematic deviations that can be corrected, while the last term is random, with a median of zero, and cannot be corrected. However, this last term can be processed employing averaging techniques.

### 4.2. Arduino Clock Drift Measurement

Equation (1) can be simplified considering that the Arduino device does not have to be synchronised with any reference time to perform the detonation time interval measurements. Having the first term null, *t*_0_ = 0, we obtain Equation (2).

(2) $$\varepsilon_{t}(t)\;=\;\left(\phi +M\cdot \left(T^{{}^{\circ}}-T_{ref}^{{}^{\circ}}\right)-1\right)\cdot t+D\cdot t^{2}+\sigma_{x}(t)$$

This means that, to know the error in the time measured by the Arduino, it must be determined how much the real frequency of the ceramic oscillator varies from its nominal value, considering the manufacturing tolerance and the external environmental factors.

#### 4.2.1. Tolerance Measurement

Tolerance error is inherent in any manufacturing process and it expresses the difference between the nominal and actual value of the characteristics of a material or product. The nominal frequency of the ceramic oscillator, controlling the clock of the Arduino microcontroller, is 16 MHz, with an accuracy of around ±0.5% at 25 °C [31]. Table 2 summarises the characteristics of the CSTCE16M0V53 oscillator on the Arduino Mega 2560 board [32].

**Figure 6:**
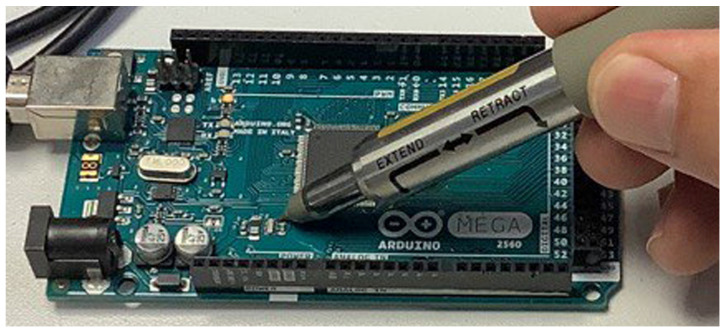
High-impedance probe measurement of the actual frequency of the ceramic oscillator.

To know the real frequency of the oscillator, *f_r_*, and be able to correct the clock signal of the microcontroller, the frequency has been measured using a Rhode&Schwarz high-impedance probe, Figure 6. Before the measurement, the board has been in a room temperature at 25 °C for one hour, being the reference temperature for the measurement of the tolerance.

**Figure 7:**
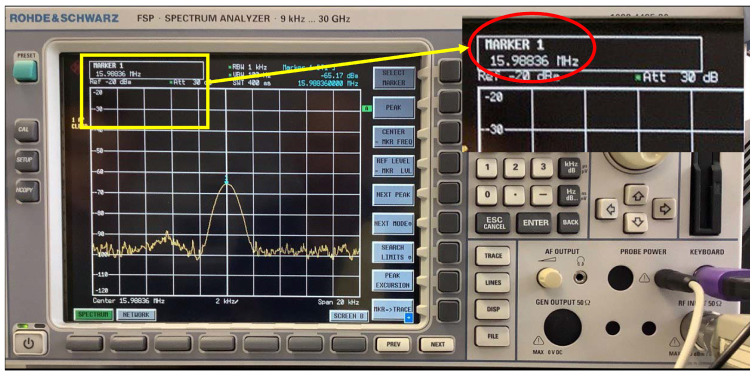
Measurement of the actual frequency of the ceramic oscillator CSTCE16M0V53. Nominal frequency: 16 MHz. Real frequency: 15.98836 MHz. Temperature: 25 °C.

Results obtained show that the ceramic oscillator of the plate used has an oscillation frequency of 15.98836 MHz at 25 °C, i.e., −727.5 ppm, with respect to its nominal value and, therefore, is within the tolerance ± 5000 ppm specified by the manufacturer. The measured frequency is shown in Figure 7.

#### 4.2.2. Frequency and Temperature Stability

**Figure 8:**
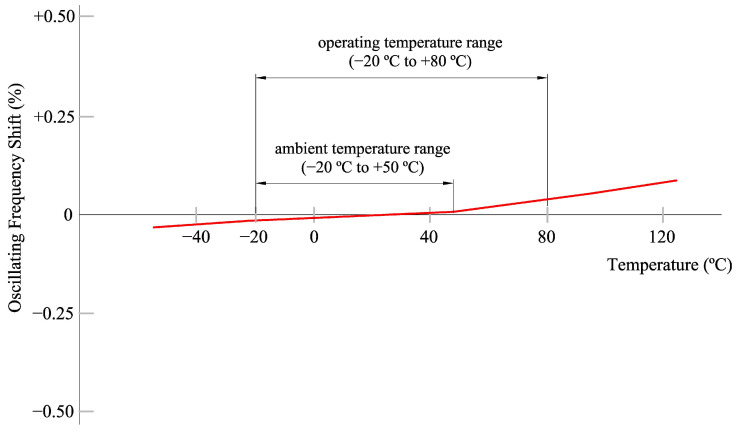
Frequency variation concerning temperature for the ceramic oscillator CSTCE_V(_A).

The frequency variation concerning temperature, for the CSTCE_V(_A) ceramic oscillator, is shown in Figure 8, adapted from catalog Cat.No.P16E-16 from Murata Manufacturing Co., Ltd., available at [32]. The oscillator is very stable to frequency change because of temperature, with a linear variation in the range of −20 °C to +50 °C with a slope of M = 3 ppm/°C. This temperature range is quite large and covers almost any extreme environmental situation in which the device would operate. The temperature range of the other components is higher and, therefore, it is not a problem for the system proposed.

In general, this correction is several orders of magnitude lower than the required equipment accuracy of 0.1 ms and, therefore, it can be neglected. Only in the case of measurements over long time intervals and at extreme temperatures could one consider taking this term into account.

#### 4.2.3. Frequency Stability against Ageing

When an oscillator changes its frequency over time, with constant environmental and system conditions, it is usually ageing [33]. The typical ageing specification for ceramic oscillators is a maximum of 0.3% per decade [31]. Age drift is related to the degradation of materials over the years. This term would only be significant if very long periods elapsed between measurements, in the order of several years or decades. Therefore, given that the time that elapsed between the measurement of the oscillator tolerance and the tests performed was only a few weeks, the *Dt*^2^ term is completely negligible in Equation (2).

#### 4.2.4. Stochastic Error

The last term from Equation (2) is a zero-median stochastic error, which cannot be corrected [34]. However, its effect can be compensated using averaging techniques. Each pulse of the periodic signal generated by the oscillator is affected by a random frequency variation, Figure 5, due to noise in the signal. However, the noise spectrum is symmetrical on both sides of the actual frequency and, thus, its mean value will tend to be zero, obtaining a negligible term for the time intervals in which measurements are made in the studies.

### 4.3. Time Correction

Once the non-significant tolerance terms are removed, the simplified equation to calculate the error of the microcontroller clock is gathered in Equation (3).

(3) $$\varepsilon_{t}=\left(\frac{T_{n}}{T_{r}}-1\right)\cdot t\;=\;\left(\phi -1\right)\cdot t$$

**Figure 9:**
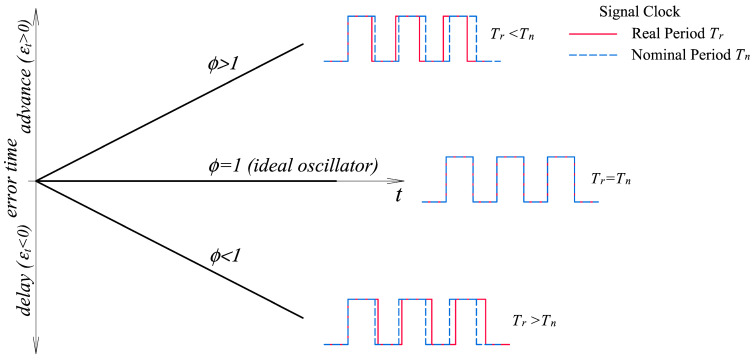
Oscillator time error as a function of the ratio of the actual period of the oscillator, *T_r_*, to the nominal period, *T_n_*.

This equation is depicted in Figure 9. In the case of an ideal oscillator, *ϕ* = 1, no time drift occurs and no correction is necessary. If the actual period of the oscillator differs from the nominal one, then *ϕ* ≠ 1 and the microcontroller clock will be advanced or delayed depending on whether *f* is greater or less than unity, respectively.

Knowing the error of the time *e_t_*, it is possible to correct the time drift measured by the Arduino device by means of Equation (4).

*t_c_* = *t_m_* (1 − *e_t_*/10^6^) (4)

*t_c_*: drift corrected time;
*t_m_*: time measured by the microcontroller clock;
*e_t_*: oscillator time error, in ppm.

This adjustment has been introduced in the control software, so that subsequent measurements incorporate this correction.

### 4.4. Oscilloscope Operational Pre-Testing

**Figure 10:**
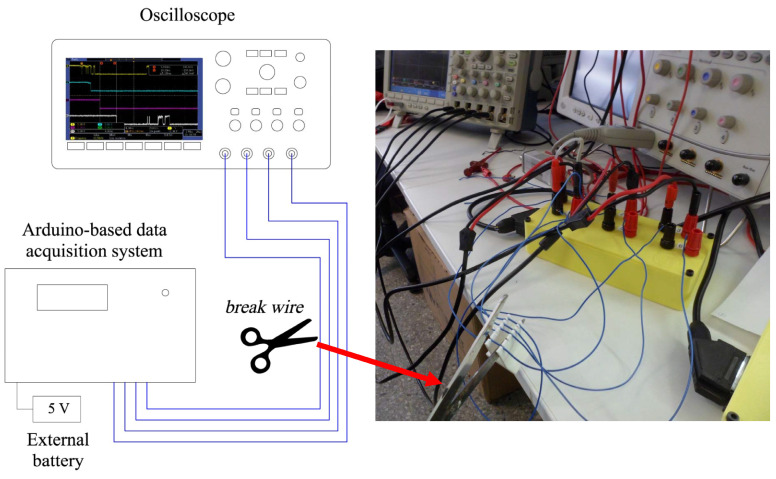
Conceptual scheme of the digital oscilloscope test.

Once it has been defined the applicable correction to the times measured by the device, its operation was checked through carrying out a test, consisting of measuring the cutting times of four signal cables with a four-channel Tektronix DPO 3054 digital oscilloscope using scissors, Figure 10. This test was used to verify the correct operation of the hardware, programmed code and the connection protocol between the oscilloscope and Arduino, which will be used in the field test with real detonators.

Wires of the measuring circuits have been placed 1 mm to 10 mm apart from each other with the help of a plastic holder. Different break times can be obtained through varying the spacing between wires and the speed at which they are cut. The measured time intervals are between 2 ms and 1 s, covering the usual range of detonation times between holes in open pit mining, underground mining and civil works.

### 4.5. Field Test

A field test was carried out to check the operation of the equipment under actual conditions. It consisted of measuring the detonation intervals of 100 non-electric detonators, with the signal cable breaking due to the effect of an explosion. The detonators have been triggered in groups of four, due to the limitation imposed by the number of channels of the oscilloscope, recording the analog signal of all the wire breaks. Once the drift of the measured times has been corrected, employing Equation (4), both results have been compared, validating the operation of the equipment in actual conditions.

#### 4.5.1. Test Location

**Figure 11:**
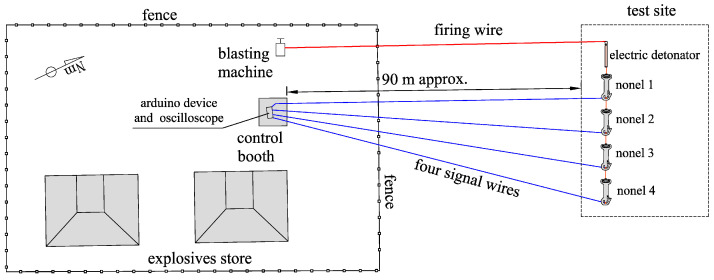
Diagram of the test site.

Tests were carried out in the vicinity of an explosives depot, which has a control hut that allows the oscilloscope to be located in adequate environmental conditions, as well as having a 220 V/50 Hz power supply. From this hut, the four bipolar signal cables have been extended to a safety distance of 90 m, as well as the firing cable of the electric detonator that initiates the shock wave transmission tube of the non-electric detonators. A diagram of the test site is shown in Figure 11.

#### 4.5.2. Test Preparation

**Figure 12:**
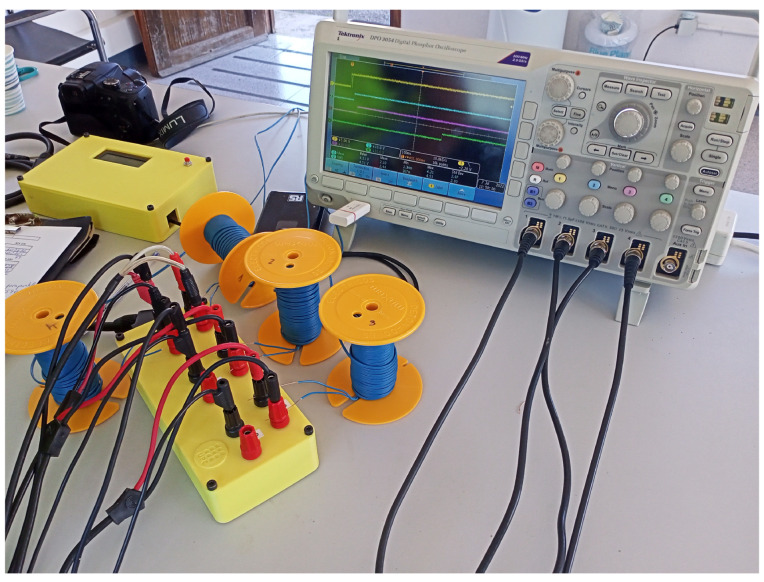
Setup to measure the detonation times.

The measurement circuits have been connected to a four-channel digital oscilloscope Tektronix DPO 3054, 500 MHz and to the Arduino, as shown in Figure 12.

**Figure 13:**
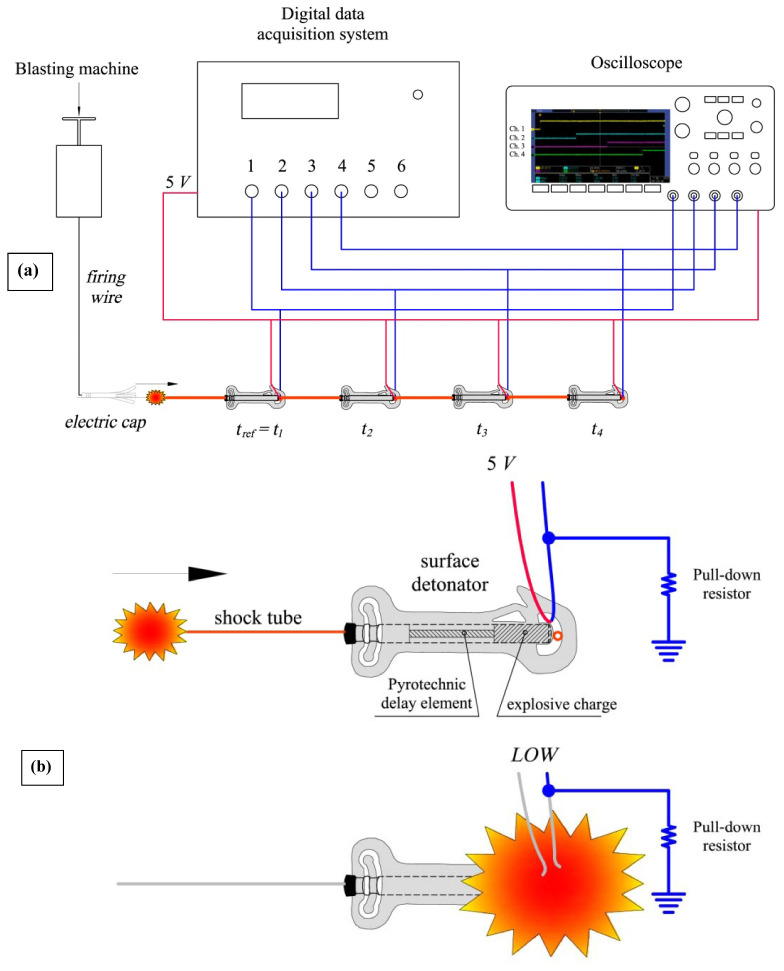
(**a**) Conceptual diagram of connection to the oscilloscope and measuring equipment for simultaneous recording of the same wire break event. (**b**) Surface detonator with signal wire.

The details of a surface detonator with signal wire to measure the detonation instant *t*_1_…*t*_4_ are displayed in Figure 13, calculating the three detonation intervals, *t*_2_–*t*_1_, *t*_3_–*t*_2_ and *t*_4_–*t*_3_, according to the time difference. The oscilloscope sampling rate is up to 2.5 Giga samples per second on all analog channels. This allows recording the step from 5 V to 0 V of each circuit, with an order of magnitude four times higher than the resolution of the function *micros()*, which is four microseconds.

**Figure 14:**
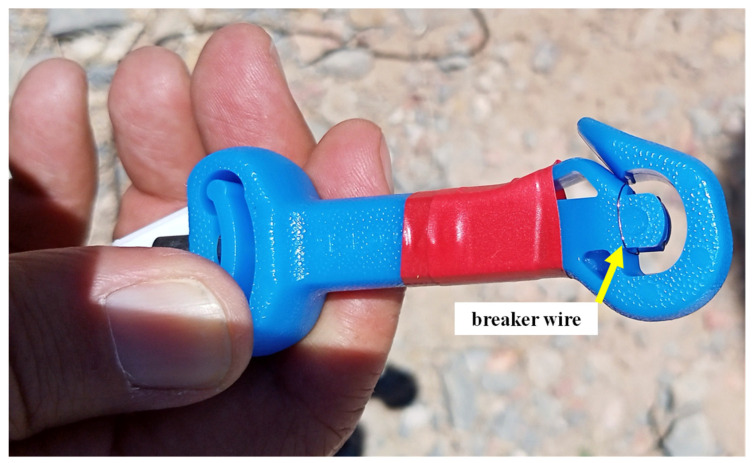
Surface detonator with 1x ø 0.19 mm copper break wire.

**Figure 15:**
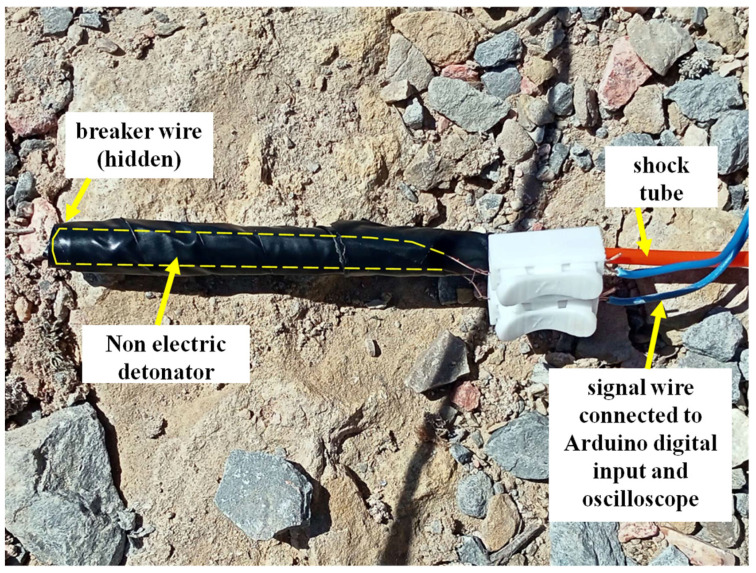
The copper break wire is attached to the end of the blasting cap containing the pentrite charge.

The 100 surface connectors/detonators were triggered in groups of four since it is a four-channel oscilloscope. Twenty-five tests were performed, covering the full range of micro time delay offered by the manufacturer, between 9 ms and 750 ms. The sets tested are shown in Table 3. The break wire has been taped to the surface connector or the detonator, as shown in Figure 14 and Figure 15.

**Figure 16:**
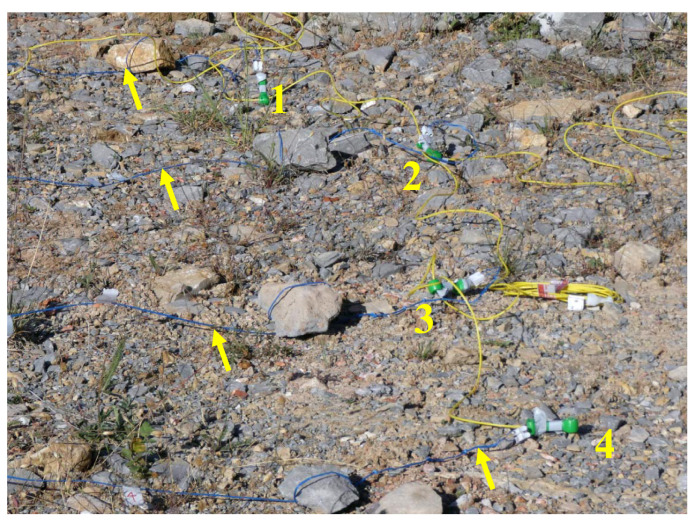
Set of four surface connectors ready for testing. The arrows indicate the signal cable.

In all tests, a length of one metre of transmission tube was left between the detonators, Figure 16. Considering that the velocity of the shock wave travels at 2000 m/s inside the tube, a delay of 0.5 ms is introduced to the detonation time of the surface connector/detonator. Since the same event is simultaneously measured using the oscilloscope and the Arduino, this delay does not influence the times obtained for the validation of the equipment.

#### 4.5.3. Temperature Correction

**Figure 17:**
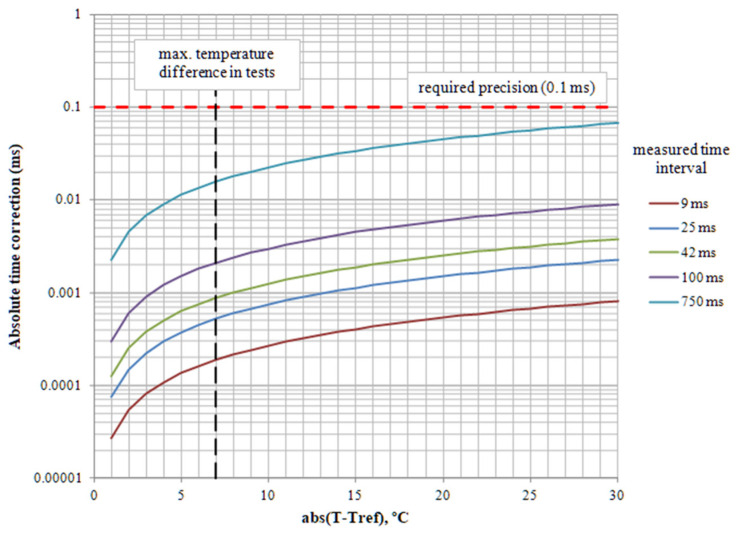
Correction time for frequency variation concerning temperature for the usual time intervals of non-electric detonators.

The temperature is 18 °C at the beginning of the tests and 27 °C at the end, so the maximum temperature difference with respect to *Tref* = 25 °C is 7 °C. For this reason, the correction of the time drift due to temperature has been omitted, as it has very low values, between 0.0002 and 0.015 ms for a coefficient of variation *M* = 3 ppm/°C (Figure 17).

## 5. Field Measurement Results

The total number of time records measured using the oscilloscope and the Arduino device is shown in Table 4. The three intervals of test 2 were not measured due to a connection failure in the devices, whereas test 5 failed due to the signal cable failing to break, being the only failure among the 100 detonators fired. In this regard, the copper wire must be slightly tensioned in the area where the explosive charge is located so that the breakage is easier. The last interval of test 13 was not recorded by the oscilloscope because it was outside the programmed recording window. Overall, 70 out of 75 possible detonation intervals were measured and compared.

**Figure 18:**
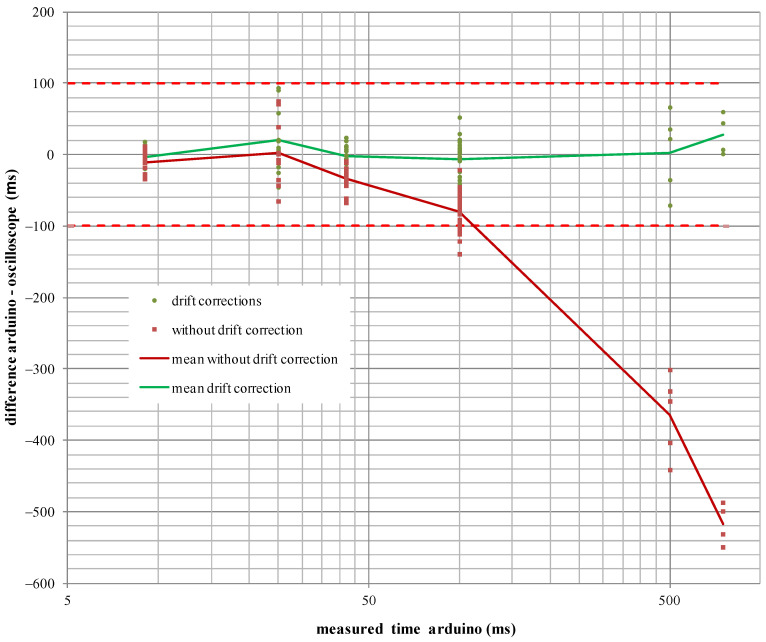
Time differences obtained between the Arduino device and the oscilloscope, without applying drift correction (red line) and applying drift correction (green line).

Results show how the average relative error of the unadjusted times (−725 ppm) is virtually identical to the measurement using a high-impedance probe of the oscillator tolerance (−727.5 ppm), confirming the accuracy of the microcontroller’s clock drift determination. The times measured using the Arduino device improve substantially when the Equation (4) setting is applied, with the average relative error going from −725 ppm to an error of only 2 ppm. The time differences are shown in Figure 18, where it can be seen that, once the oscillator drift correction is applied, the differences are smaller than the minimum required accuracy, 0.1 ms, needed to measure the dispersion times of pyrotechnic detonators.

Regarding the influence of temperature and signal noise, it is confirmed that both can be neglected for the required level of accuracy. The measurement of detonation times using the Arduino’s digital pins is faster and more direct than using the oscilloscope, as no manual operations are required. Additionally, errors such as the one in test 13, in which the Arduino device recorded the instant of breakage of the cable, occurred because the detonation moment was accidentally outside the programmed recording window due to the dispersion of the pyrotechnic delay detoantors. The proposed device can measure up to six detonation instants, overcoming the oscilloscope’s limitation of four analog channels. Future prototypes could easily extend this number as there are still digital pins available, making the measurement of detonation time dispersion from production blasts faster and more efficient.

## 6. Conclusions

The device proposed in this study, based on the open-source Arduino platform, has shown to be a good option to measure the variation of detonator delay times. Results obtained show the feasibility of using low-cost equipment to make this type of measurement and to know the detonation instant of surface connectors and bottom-hole detonators. In addition, the simplicity of using it in situ, without interfering with mining or civil work, and the possibility to make future changes, adaptations or improvements allow new potential users compared to other commercial systems.

The ceramic oscillator that controls the time of the Arduino’s internal clock has a tolerance that must be known to correct the internal clock signal properly. The real frequency of the oscillator used has been measured using a high-impedance probe, finding a difference of 727.5 ppm with respect to its nominal value of 16 MHz. This factor is the main error source to be corrected, being preponderant concerning the deviation caused by temperature changes, aging, and stochastic errors, which are several orders of magnitude below the minimum required accuracy of 0.1 ms.

The field test carried out using 100 non-electric detonators for open-pit blasting, with nominal times between 9 and 750 ms, corroborates the accuracy and good performance of the equipment. Once the oscillator tolerance correction has been applied, absolute differences are less than 0.1 ms between the time measured using an oscilloscope, with a sampling frequency of 2.5 Giga samples per second, and the measures from the Arduino.

## Figures and Tables

**Table 1:** Comparison of the existing techniques.

	Acustic Sensors	VoD Measurement Equipment	High-Speed Camera	Oscilloscope. Open-Circuit Probe	Arduino Device
**pros**	Simple.	Robust equipment designed for production blasting measurements.	No connection is necessary for the measurements. Additionally, it has other interesting applications such as measuring the rock displacement velocity.	High accuracy.	Simple and inexpensive.
**cons**	Requires the same distance between detonators and the sensor. Not suitable for production blasting.	Expensive and it cannot be used for surface connectors measurement.	Very expensive. It cannot be used for measurements of detonators inside blast holes.	Expensive. Delicate equipment for use in field conditions.	It is necessary to make connections and lay cables for measurements.

**Table 2:** Characteristics of the ceramic oscillator CSTCE16M0V53 of the Arduino MEGA 2560 board.

Manufacturer	Murata
Part Number	CSTCE16M0V53-R0
Composition	Ceramic
Frequency	16 MHz
Frequency Tolerance	±0.5%
Max Operating Temperature	80 °C
Min Operating Temperature	−20 °C

**Table 3:** Surface connectors and detonators used in the validation tests.

	Circuit Number 1 (Trigger)	Circuit Number 2	Circuit Number 3	Circuit Number 4	Number of Tests
set 1	S-9	S-9	S-9	S-9	5
set 2	S-42	S-42	S-42	S-42	5
set 3	S-100	S-100	S-100	S-100	5
set 4	S-100	S-100	S-100	I-750	5
set 5	S-25	S-25	S-25	I-500	5

S-X: Surface detonator, I-X: In-hole detonator, X: nominal time (ms).

**Table 4:** Set of tests and times measured using the oscilloscope and the Arduino device.

Test Num.	Test Set	Measured Chanels	*t_m_* Arduino Measured Interval Time (ms)	*t_c_* Adjusted Using Equation (4) (ms)	*t* Oscilloscope (ms)	*t_m_*-*t* Difference without Adjustment (ms)	Relative Error without Adjustment (ppm)	Relative Error with Adjustment Equation (4) (ppm)
1	set 1	1/2	10,892	10,900	10,887	5	459	1187
		2/3	8720	8726	8754	−34	−3884	−3159
		3/4	10,484	10,492	10,490	−6	−572	155
2	set 1	1/2	(*)	---	(*)	---	---	---
3	set 1	1/2	8904	8910	8912	−8	−898	−171
		2/3	10,500	10,508	10,500	0	0	728
		3/4	10,616	10,624	10,628	−12	−1129	−402
4	set 1	1/2	11,080	11,088	11,076	4	361	1089
		2/3	9596	9603	9608	−12	−1249	−522
		3/4	10,789	10,797	10,808	−19	−1758	−1032
5	set 2	1/2	40,616	40,646	40,678	−62	−1524	−798
		2/3	42,096	42,127	42,120	−24	−570	157
		3/4	(**)	---	---	---	---	---
6	set 2	1/2	39,552	39,581	39,562	−10	−253	475
		2/3	42,052	42,083	42,064	−12	−285	442
		3/4	42,884	42,915	42,952	−68	−1583	−857
7	set 2	1/2	42,288	42,319	42,350	−62	−1464	−738
		2/3	41,640	41,670	41,684	−44	−1056	−329
		3/4	45,524	45,557	45,534	−10	−220	508
8	set 2	1/2	41,380	41,410	41,420	−40	−966	−239
		2/3	46,112	46,146	46,134	−22	−477	250
		3/4	41,128	41,158	41,164	−36	−875	−148
9	set 3	1/2	100,144	100,217	100,200	−56	−559	168
		2/3	96,952	97,023	97,032	−80	−824	−98
		3/4	97,812	97,883	97,876	−64	−654	73
10	set 3	1/2	104,748	104,824	104,870	−122	−1163	−437
		2/3	101,824	101,898	101,880	−56	−550	177
		3/4	101,848	101,922	101,920	−72	−706	21
11	set 3	1/2	102,304	102,378	102,350	−46	−449	278
		2/3	100,880	100,953	100,990	−110	−1089	−363
		3/4	102,676	102,751	102,760	−84	−817	−91
12	set 3	1/2	99,740	99,813	99,808	−68	−681	46
		2/3	99,936	100,009	100,000	−64	−640	87
		3/4	102,228	102,302	102,310	−82	−801	−75
13	set 4	1/2	98,976	99,048	99,080	−104	−1050	−323
		2/3	96,832	96,902	96,890	−58	−599	128
		3/4	754,460	755,009	(***)	---	---	---
14	set 4	1/2	101,380	101,454	101,520	−140	−1379	−653
		2/3	98,112	98,183	98,190	−78	−794	−67
		3/4	752,112	752,659	752,600	−488	−648	79
15	set 4	1/2	97,288	97,359	97,400	−112	−1150	−423
		2/3	99,448	99,520	99,500	−52	−523	205
		3/4	756,560	757,110	757,110	−550	−726	1
16	set 4	1/2	100,928	101,001	100,950	−22	−218	509
		2/3	94,708	94,777	94,800	−92	−970	−244
		3/4	739,868	740,406	740,400	−532	−719	8
17	set 5	1/2	25,300	25,318	25,300	0	0	727
		2/3	23,492	23,509	23,500	−8	−340	387
		3/4	505,596	505,964	506,000	−404	−798	−71
18	set 5	1/2	25,864	25,883	25,790	74	2869	3599
		2/3	26,760	26,779	26,690	70	2623	3352
		3/4	508,968	509,338	509,410	−442	−868	−141
19	set 5	1/2	24,992	25,010	24,990	2	80	808
		2/3	24,584	24,602	24,620	−36	−1462	−736
		3/4	504,248	504,615	504,580	−332	−658	69
20	set 5	1/2	26,768	26,787	26,730	38	1422	2150
		2/3	27,004	27,024	27,070	−66	−2438	−1712
		3/4	505,288	505,656	505,590	−302	−597	130
21	set 1	1/2	10,480	10,488	10,508	−28	−2665	−1939
		2/3	8920	8926	8909	11	1212	1941
		3/4	9324	9331	9359	−35	−3718	−2994
22	set 2	1/2	41,996	42,027	42,028	−32	−761	−34
		2/3	41,068	41,098	41,088	−20	−487	240
		3/4	41,628	41,658	41,654	−26	−624	103
23	set 3	1/2	100,772	100,845	100,870	−98	−972	−245
		2/3	100,892	100,965	100,970	−78	−773	−46
		3/4	99,716	99,789	99,792	−76	−762	−35
24	set 4	1/2	91,192	91,258	91,290	−98	−1074	−347
		2/3	98,520	98,592	98,590	−70	−710	17
		3/4	747,000	747,543	747,500	−500	−669	58
25	set 5	1/2	25,368	25,386	25,380	−12	−473	254
		2/3	24,796	24,814	24,840	−44	−1771	−1045
		3/4	504,864	505,231	505,210	−346	−685	42
						**Mean**	**−725**	**2**
						**St. desv**	**1011**	**1012**

(*) Error connection; (**) no wire breakage; (***) time value out screen.

## Data Availability

Data unavailable do to privacy restrictions.
